# Quantitative assay to analyze neutralization and inhibition of authentic Middle East respiratory syndrome coronavirus

**DOI:** 10.1007/s00430-024-00789-w

**Published:** 2024-05-09

**Authors:** Helena Müller-Kräuter, Jolanda Mezzacapo, Michael Klüver, Sara Baumgart, Dirk Becker, Anahita Fathi, Sebastian Pfeiffer, Verena Krähling

**Affiliations:** 1 Institute of Virology, Philipps University Marburg, Hans-Meerwein Str. 2, 35043 Marburg, Germany; 2 German Center for Infection Research (DZIF), Partner Site Gießen-Marburg-Langen, Marburg, Germany; 3 Institute for Infection Research and Vaccine Development, University Medical Center Hamburg-Eppendorf, Hamburg, Germany; 4 German Center for Infection Research (DZIF), Partner Site Hamburg-Lübeck-Borstel-Riems, Hamburg, Germany

**Keywords:** MERS-CoV, Middle East respiratory syndrome coronavirus, Neutralization, MNA, VNT, Vaccine

## Abstract

To date, there is no licensed vaccine for Middle East respiratory syndrome coronavirus (MERS-CoV). Therefore, MERS-CoV is one of the diseases targeted by the Coalition for Epidemic Preparedness Innovations (CEPI) vaccine development programs and has been classified as a priority disease by the World Health Organization (WHO). An important measure of vaccine immunogenicity and antibody functionality is the detection of virus-neutralizing antibodies. We have developed and optimized a microneutralization assay (MNA) using authentic MERS-CoV and standardized automatic counting of virus foci. Compared to our standard virus neutralization assay, the MNA showed improved sensitivity when analyzing 30 human sera with good correlation of results (Spearman’s correlation coefficient *r* = 0.8917, *p* value < 0.0001). It is important to use standardized materials, such as the WHO international standard (IS) for anti-MERS-CoV immunoglobulin G, to compare the results from clinical trials worldwide. Therefore, in addition to the neutralizing titers (NT_50_ = 1384, NT_80_ = 384), we determined the IC_50_ and IC_80_ of WHO IS in our MNA to be 0.67 IU/ml and 2.6 IU/ml, respectively. Overall, the established MNA is well suited to reliably quantify vaccine-induced neutralizing antibodies with high sensitivity.

**Supplementary Information:**

The online version contains supplementary material available at 10.1007/s00430-024-00789-w.

## Introduction

Middle East respiratory syndrome coronavirus (MERS-CoV) infections are usually severe and associated with a high mortality rate of 10–35% [1]. To date, there is no approved vaccine against MERS-CoV, but several vaccine candidates are already being tested in clinical trials [2–5]. The humoral immunogenicity of vaccines is analyzed by detecting virus-binding and virus-neutralizing antibodies. Enzyme-linked immunosorbent assays (ELISAs) are used to quantify antibodies and virus neutralization tests (VNTs) to confirm antibody functionality. Although time-consuming and expensive, neutralization tests using authentic viruses remain the gold standard [6]. We use the microscopic evaluation of MERS-CoV-induced cytopathic effects (CPE) in cell culture to monitor MERS-CoV-neutralizing antibodies [2, 4, 5]. This method is well suited as it can be used to reliably analyze 100% inhibition of MERS-CoV (VNT_100_). However, this method is subject to some operator-dependent variability and is generally considered to be less sensitive compared to assays that assess 50% inhibition of the virus [6]. To address these limitations and complement our VNT_100_, we wanted to develop another MERS-CoV neutralization test based on publications for SARS-CoV-2 [7, 8].

In the present study, we describe the establishment of a MERS-CoV-specific microneutralization assay (MNA) and compare the developed assay to our standard VNT_100_ to detect virus-neutralizing antibodies [2, 4, 5]. Furthermore, we show that the assay can also be used for inhibitor testing against MERS-CoV.

## Materials and methods

### Cells and viruses

Vero C1008 cells (ATCC CRL-1586) and HuH7 cells (fully matching the STR reference profile of HuH-7) were cultured as described elsewhere [9]. Vero C1008 and HuH7 cells were authenticated in 2016 by DNA profiling of eight highly polymorphic regions of short tandem repeats by the “Leibniz-Institut DSMZ (Deutsche Sammlung von Mikroorganismen und Zellkulturen) GmbH” and are proven to be free of mycoplasma through regular testing. MERS-CoV (EMC/2012) (GenBank: NC_019843.3) was propagated and titrated in Vero C1008 cells (2.3 × 10^7^ plaque-forming units (PFU/ml) and in HuH7 cells (1.23 × 10^7^ PFU/ml). The virus sequence was determined by next-generation sequencing. The consensus sequence obtained showed three mutations that resulted in amino acid (AA) changes compared to the reference sequence. Two of them are in the spike protein: arginine instead of glycine (AA 94) and alanine instead of valine (AA 1026), and one is in ORF5, where tryptophan 108 is converted to a stop codon.

### Antibodies, serum samples, and inhibitors

An anti-MERS-CoV spike protein antibody (m336, Nanjing Mingyan Biotechnology Co., Ltd.) neutralizing MERS-CoV with high potency [10, 11] diluted in human serum (Sigma-Aldrich, H5667) and the 1st International Standard for anti-MERS-CoV immunoglobulin G (human) (NIBSC code: 19/178) (WHO IS) were used. This study used serum samples collected from an anti-MERS-CoV vaccine trial (phase Ib clinical trial of MVA-MERS-S_DF-1 in healthy individuals, NCT04119440). The pan-coronavirus inhibitor EK1C4 (InVivoGen, #inh-ek1c4) was dissolved in DMSO (100 µM). Specific staining of MERS-CoV-infected cells was performed with a rabbit anti-MERS-CoV nucleoprotein antibody (1:2000, Sino Biological, #40068-RP02) and polyclonal swine anti-rabbit immunoglobulins/HRP (1:1000, Agilent Dako, #P021702-2).

### MERS-CoV neutralization assay (VNT_100_)

The MERS-CoV neutralization assay was performed as previously described [2]. Briefly, sera were serially diluted and incubated with 100 PFU of MERS-CoV for 1 h. Thereafter, HuH7 cells were added to the mixture of serum and virus. CPE was evaluated at day 4 post infection. Neutralization was defined as the absence of CPE. Neutralization titers of three replicates were calculated as geometric means (reciprocal value). The lower detection limit of the assay is 8. Dulbecco’s modified Eagle’s medium (DMEM) supplemented with 3% fetal calf serum (FCS), penicillin (50 U/ml), streptomycin (50 μg/ml) (*P*/*S*), and glutamine (2 mM) (*Q*) was used in the assay.

### Microneutralization assay (MNA)

All serum samples tested were complement inactivated at 56 °C for 30 min. On each 96-well plate, a no-virus control (NVC), a virus-only control (VOC), and the anti-MERS-CoV spike m336 antibody served as controls (Online Resource, Fig. 1). Serum samples (initial dilution 1:10) and m336 (initial concentration 200 ng/ml) were serially diluted 1:3 in 80 µl DMEM supplemented with 1% FCS, *P*/*S*, and *Q* (DMEM 1%++). To generate 150 virus foci per well (Online Resource, Fig. 2), 80 µl of diluted MERS-CoV was added to each well, except for the NVC, where 80 µl DMEM 1%++ was added instead. Considering the virus titer of 2.3 × 10^7^ PFU/ml, approximately 2000 PFUs are used to infect one well. MERS-CoV was incubated with the sera/antibody for 1 h. Then 100 µl was transferred to Vero C1008 cells (22,000 cells per well) and after another hour, the supernatant was replaced with 2% carboxymethyl cellulose (CMC; Sigma Aldrich, #C5678), 2% FCS, *P*/*S*, and *Q* in Minimal Essential Medium Eagle. After 24 h, the cells were fixed twice for 24 h with 4% paraformaldehyde to inactivate the virus and remove the plates from the BSL4. This was followed by staining and visualization of the virus foci by addition of 3,3′,5,5′-tertamethylbenzidine substrate (True Blue; Sera Care, #5510-0030). The number of foci per well was determined using ImmunoSpot S6 Ultra-V Analyzer (CTL, S6ULTRA-02-6147/Series 5). The foci reduction compared to the VOC was calculated for each serum or antibody dilution. These values were used to interpolate 50% or 80% serum neutralization titer (NT_50_ or NT_80_) or inhibitory concentration (IC_50_ or IC_80_) by four-parameter logistic (4PL) regression analysis (GraphPad Prism 9 Software, La Jolla, California).

To determine the IC_50_ of EK1C4, the MNA was modified according to Xia et al., 2020 [12]. EK1C4 was serially diluted 1:2 (initial dilution 500 nM) and analyzed twice in duplicate. The incubation time of MERS-CoV with EK1C4 and m336 was reduced from 1 h to 30 min.

## Results

We wanted to establish an MNA that reliably quantifies MERS-CoV-specific neutralizing antibodies.

**Fig. 1:**
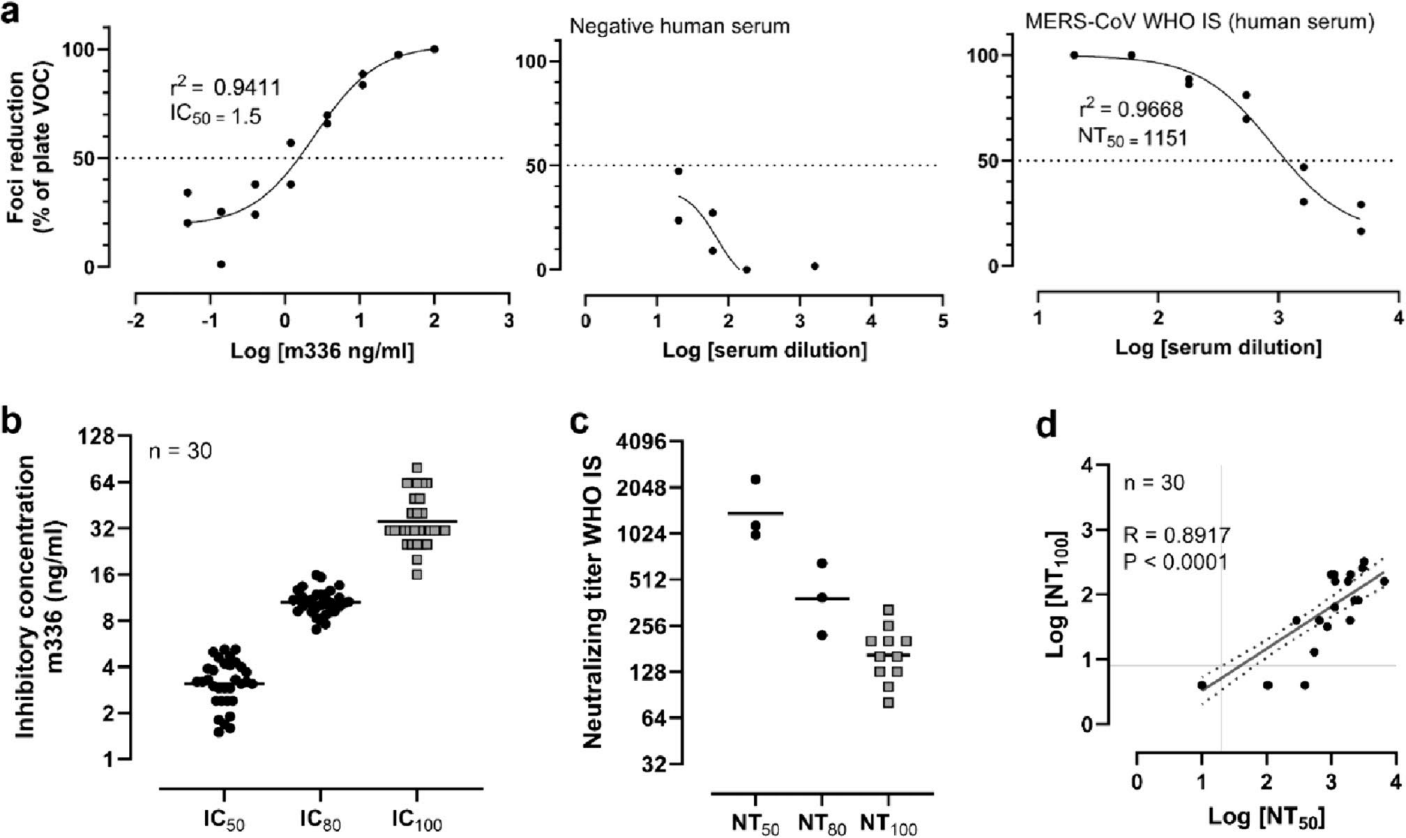
Comparison of MERS-CoV MNA to VNT_100_. **a** Exemplary inhibition curves of the antibody m336, a negative human serum, and the MERS-CoV WHO international standard for immunoglobulins (WHO IS, human serum) in the microneutralization assay (MNA). The logarithmic concentration of m336 or the logarithmic serum dilution is plotted against the foci reduction in % of the virus only control (VOC). *R*^2^ values from the 4PL regression analysis and the calculated IC_50_ values are noted on the graph. The dotted line visualizes the 50% inhibition compared to VOC. Repeated analysis of m336 (**b**) and WHO IS (**c**) in the MNA and the VNT_100_ to calculate IC_50_ and IC_80_ values (MNA) or IC_100_ values (VNT_100_). **d** Correlation of the IC_50_ values generated with the MNA and the IC_100_ values of the VNT_100_. The line reflects the best-fit linear relationship between the variables, and the dotted lines represent 95% confidence intervals. *p* and *r* values reflect two-tailed Spearman’s rank-correlation tests. The LLODs of each test are visualized by the gray lines

First, we determined that an incubation time of 24 h and 2% CMC as the viscous overlay of Vero C1008 cells were optimal conditions to generate morphologically homogenous virus foci by MERS-CoV (Online Resource, Fig. 2a). We further determined the dilution of MERS-CoV sufficient to generate approximately 150 virus foci per well as a dilution of 1:564 (Online Resource, Fig. 2b). MERS-CoV was diluted accordingly for all subsequent tests. We then established a positive control to neutralize MERS-CoV using the well-characterized antibody m336 [10] (Online Resource, Fig. 2c). In subsequent experiments, m336 was used at a 1:3 serial dilution on each test plate to indicate reproducible results (Fig. 1a).

After the MNA was established, we analyzed negative and positive serum samples for their neutralization capacity against MERS-CoV. Exemplary inhibition curves are shown in Fig. 1a. To compare the VNT_100_ to the newly established MNA, we performed repeated analyses of m336 (Fig. 1b) and WHO IS (Fig. 1c). The geometric mean IC_50_ and IC_80_ values of m336 as determined by MNA were 3.1 ng/ml (coefficient of variation (CV) 32%) and 10.5 ng/ml (CV 19%), respectively. The geometric mean inhibitory concentration to achieve 100% inhibition (IC_100_) of MERS-CoV measured by VNT_100_ was 36 ng/ml (CV 42%). As expected, MERS-CoV was inhibited by 50% using a lower concentration of m336 compared to 100% inhibition of the virus. The same dependency was found when analyzing the neutralizing capacity of the WHO IS. The NT_50_ and NT_80_ were 1384 and 384, respectively, whereas NT_100_ was 165 (Fig. 1c). In addition, 30 serum samples from MVA-MERS-CoV vaccinated or MERS-CoV antibody-negative (pre-vaccination baseline samples) volunteers were analyzed in the MNA to compare the results with existing data from the VNT_100_. In both assays, 10 of these samples tested negative and 17 of these samples tested positive for neutralizing antibodies against MERS-CoV. The three remaining samples from MVA-MERS-CoV vaccinated volunteers after two vaccinations were only tested positive in the newly developed MNA. Furthermore, the correlation analysis showed a very good correlation between the results of the two neutralization tests (Fig. 1d).

**Fig. 2:**
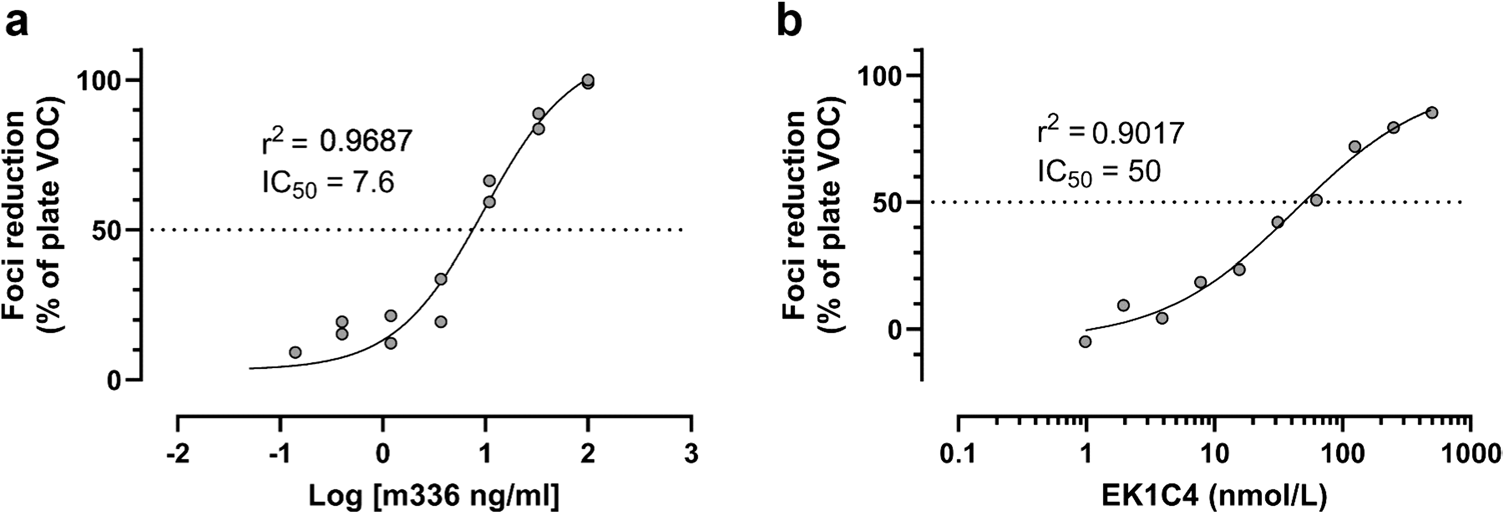
Inhibition of MERS-CoV by the pan-CoV inhibitor EK1C4. The inhibition curves of the antibody m336 (**a**) and EK1C4 (**b**) in the MNA. The logarithmic concentration of m336 or the molarity of EK1C4 is plotted against the foci reduction in % of the virus only control (VOC). *R*^2^ values from the 4PL regression analysis and the calculated IC_50_ values are noted on the graph

Finally, we wanted to test whether the developed assay can be used to analyze specific inhibitors. For this purpose, we used the pan-coronavirus fusion inhibitor EK1C4 according to Xia et al. [12]. We found that EK1C4 was able to inhibit MERS-CoV with an IC_50_ value of 50 nM in our assay. The IC_50_ value of m336 in this test was 7.6 ng/ml (Fig. 2). This differed from previous experiments and was the result of cutting the incubation time of MERS-CoV with m336 in half.

In summary, we have established a MERS-CoV MNA that can reliably quantify neutralizing antibodies. A comparison of the MNA with the VNT_100_ showed better sensitivity coupled with a good correlation of the results.

## Discussion

In the present study, we established a MERS-CoV-specific MNA using staining of virus foci to demonstrate neutralization. We compared this newly developed quantitative assay with our standard virus neutralization test (VNT_100_), in which we evaluate neutralization by an analysis of CPE. Both neutralization tests use authentic virus for multiple replication cycles and are therefore considered the gold standard as they best mimic the natural situation [6]. Neutralization assays based on pseudotypes, such as vesicular stomatitis virus or lentiviruses that contain only the foreign viral surface glycoprotein, have major limitations. They are often restricted to one viral replication cycle, and since only the major surface protein of the virus is incorporated, the expression, the density, and geometry of the protein may differ from those of authentic virus. This can affect the ability of antibodies to bind and neutralize the virus [6].

In addition to the advantages, our VNT_100_ has limitations: Due to the microscopic evaluation, it is subject to operator-dependent variability and the results cannot be easily documented. The VNT_100_ is less sensitive as the test material must contain sufficient antibodies to neutralize almost 100% of the virus used [6]. Furthermore, several replicates and thus relatively large amounts of serum are required for the analysis. By developing our new assay, we have overcome these limitations. Now less serum is required, the sensitivity has been improved, and we automatically document the results with a spot analyzer. However, this assay also has a limitation. For historical reasons, we work with MERS-CoV under BSL4 conditions, even though it is a BSL3 pathogen. Due to the time constraints of BSL4 work (4 h per entry), the plates must be inactivated and removed from the BSL4 for staining and analysis as these works would take longer than 4 h or tie up more staff. The practical procedure of the assay limits the number of samples that can be analyzed as well because in the same working time only 24 samples can be analyzed in the MNA, while 80 samples can be analyzed in the VNT_100_. In this regard, the VNT_100_ has an advantage. Since the assay is evaluated microscopically in the BSL4 and practical procedures are more rapid, a larger number of samples can be analyzed.

We have shown that the MNA is more sensitive than the VNT_100_ most likely because the IC_50_ is determined instead of the IC_100_. And this is the case even though about 2000 PFUs are used in the MNA compared to 100 PFUs in the VNT_100_ to infect the cells of a well. However, as shown by others, such a high amount of virus is required in the MNA [8] to achieve the desired amount of 100–150 virus foci. Amanat et al. have shown that approximately 10,000 TCID_50_ need be used to generate 100 foci per well in their MNA for SARS-CoV-2 [8]. Since other differences between the two assays can also contribute to the MNA being more sensitive than the VNT, such as the cell lines or the incubation time used, we have provided a detailed comparison of the main characteristics of the two tests in the supplement (Online resource, Table 1).

To compare our assays, we used a receptor-binding domain-specific monoclonal antibody, m336. The IC_50_ of m336 to neutralize authentic MERS-CoV was determined to be 70 ng/ml [10]. In our assay, the IC_50_ of m336 was determined to be 3.1 ng/ml. We cannot definitively explain this discrepancy. However, it could be due to differences in the viruses used, the experimental procedures, as well as differences in the storage and solvent of the antibody. Ying et al. used 200 TCID_50_ of a clinical isolate of MERS-CoV (not specified in more detail) and incubated it with serial dilutions of m336 for 2 h before transfer to confluent Vero cells. Evaluation was performed at 3 days post infection by microscopy of CPE. In terms of antibody stability, we have found that when m336 is stored in phosphate-buffered saline, a single freeze–thaw cycle severely impairs the function of the antibody (personal observation, VK, unpublished).

Neutralization tests are often not comparable. Therefore, it is of particular importance to use standardized materials such as the WHO IS, to be able to compare the results. In 2019, a collaborative study to investigate the comparability of serologic assays for MERS-CoV demonstrated that the use of a reference reagent, such as WHO IS, greatly improves the agreement between assays, enabling more consistent and therefore more meaningful comparisons between results [13]. Therefore, in addition to the neutralizing titers, we determined the IC_50_ and IC_80_ of WHO IS in our MNA with 0.67 IU/ml and 2.6 IU/ml, respectively. The WHO IS has been available since 2020 [14]. Unfortunately, we have not found any published results on MERS-CoV neutralization tests to compare the results of our test with other tests. Probably because tests were published before the WHO IS was available. Furthermore, we analyzed the inhibitory effect of the pan-coronavirus inhibitor EK1C4 in our MNA. Xia et al., 2020, showed that EK1C4 can inhibit multiple CoVs, including MERS-CoV. They have shown that MERS-CoV infection was inhibited with an IC_50_ of 4.2 nM [12]. In our assay, the IC_50_ was determined to be 50 nM. Xia et al. used a plaque reduction neutralization test with 100 TCID_50_ MERS-CoV per well for 72 h of infection of VeroE6 cells. In comparison, we use 2000 PFU for 24 h. This leads us to assume that the results were so different because Xia et al. used fewer virus.

We demonstrated that the correlation between our assays is good and that both are suitable to determine the neutralizing capacity of serum samples as well as antibodies. As both assays have limitations, a combination of both assays is optimal for the reliable detection of vaccine-induced neutralizing antibodies in clinical trials.

## Supplementary Information

Below is the link to the electronic supplementary material.

Supplementary file1 (PDF 843 kb)

## Data Availability

All data generated or analyzed during this study are included in this published article and its supplementary information files.
