# Supplementary material for: Quantitative assay to analyze neutralization and inhibition of authentic Middle East respiratory syndrome coronavirus
Source: Med Microbiol Immunol. 2024 May 9;213(1):6. doi: 10.1007/s00430-024-00789-w (PMC11082005; doi:10.1007/s00430-024-00789-w)
Supplement: Supplementary file 1 — Supplementary file1 (PDF 843 kb) [file 430_2024_789_MOESM1_ESM.pdf]

Medical Microbiology and Immunology

Quantitative assay to analyze neutralization and inhibition of authentic Middle East respiratory syndrome coronavirus

Helena Müller-Kräuter[1,*], Jolanda Mezzacapo[1,*], Michael Klüver[1,2], Sara Baumgart[1], Dirk Becker[1,2], Anahita Fathi[3,4], Sebastian Pfeiffer[1], Verena Krähling[1,2]

[1] Institute of Virology, Philipps University Marburg, Marburg, Germany

[2] German Center for Infection Research (DZIF), Partner Site Gießen-Marburg-Langen, Marburg, Germany

[3] University Medical Center Hamburg-Eppendorf, Institute for Infection Research and Vaccine Development, Hamburg, Germany

[4] German Center for Infection Research (DZIF), Partner site Hamburg-Lübeck-Borstel-Riems, Hamburg, Germany

* These authors contributed equally to the study

Corresponding author: Verena Krähling, email: kraehliv@staff.uni-marburg.de

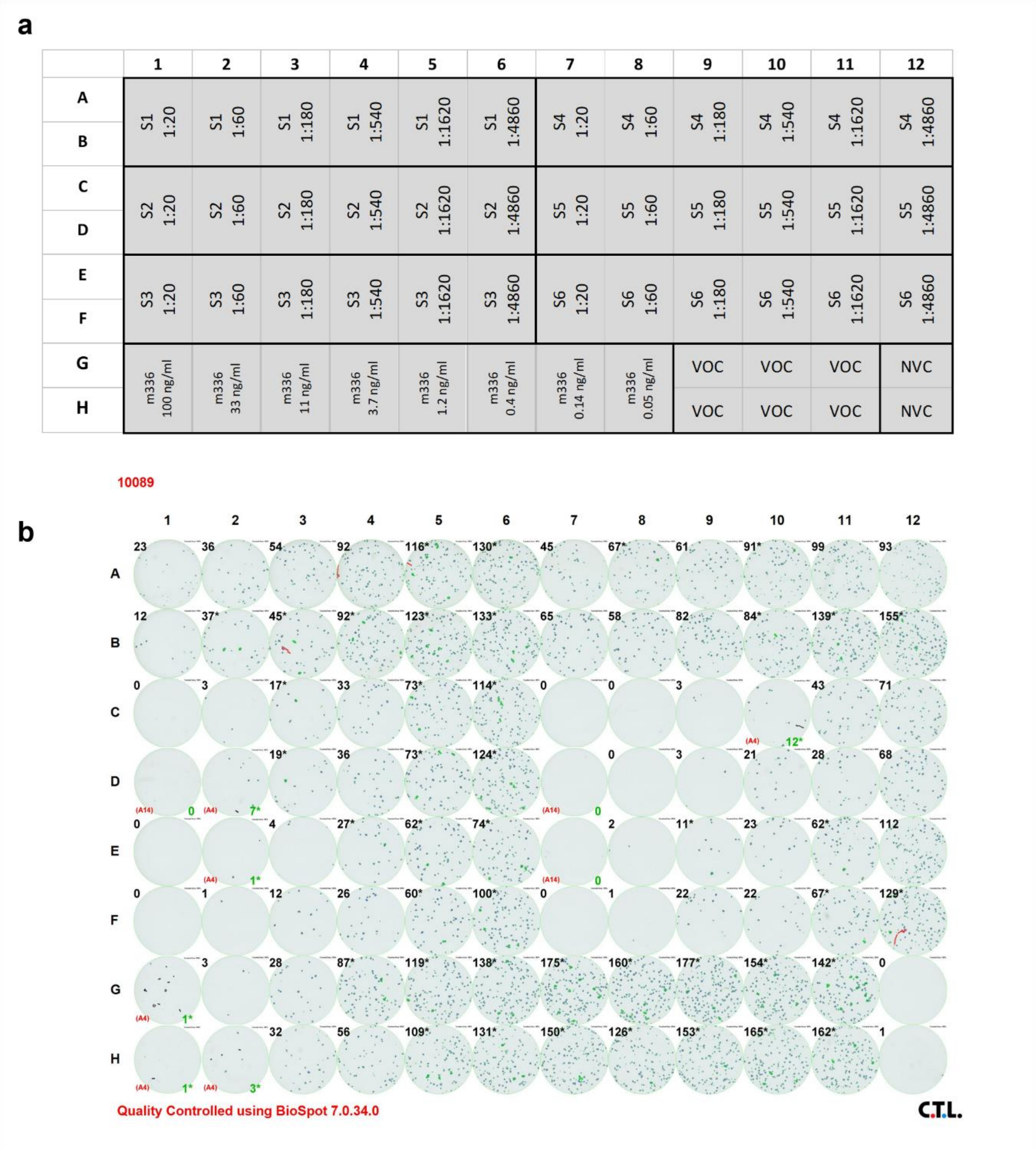

Supplementary Fig. 1 Microneutralization assay plate layout

a) A typical microneutralization assay (MNA) plate layout is shown. Rows G and H contain the controls: no-virus control (NVC), virus-only control (VOC), and the anti-MERS-CoV spike m336 antibody in eight dilutions starting with an initial concentration of 100 ng/ml. The serum samples to be tested (S1 to S6) as well as m336 are analyzed in duplicate in a dilution series of 1:3. The initial dilution of serum samples is 1:20. **b)** Exemplary results of a 96-well plate in the MNA.

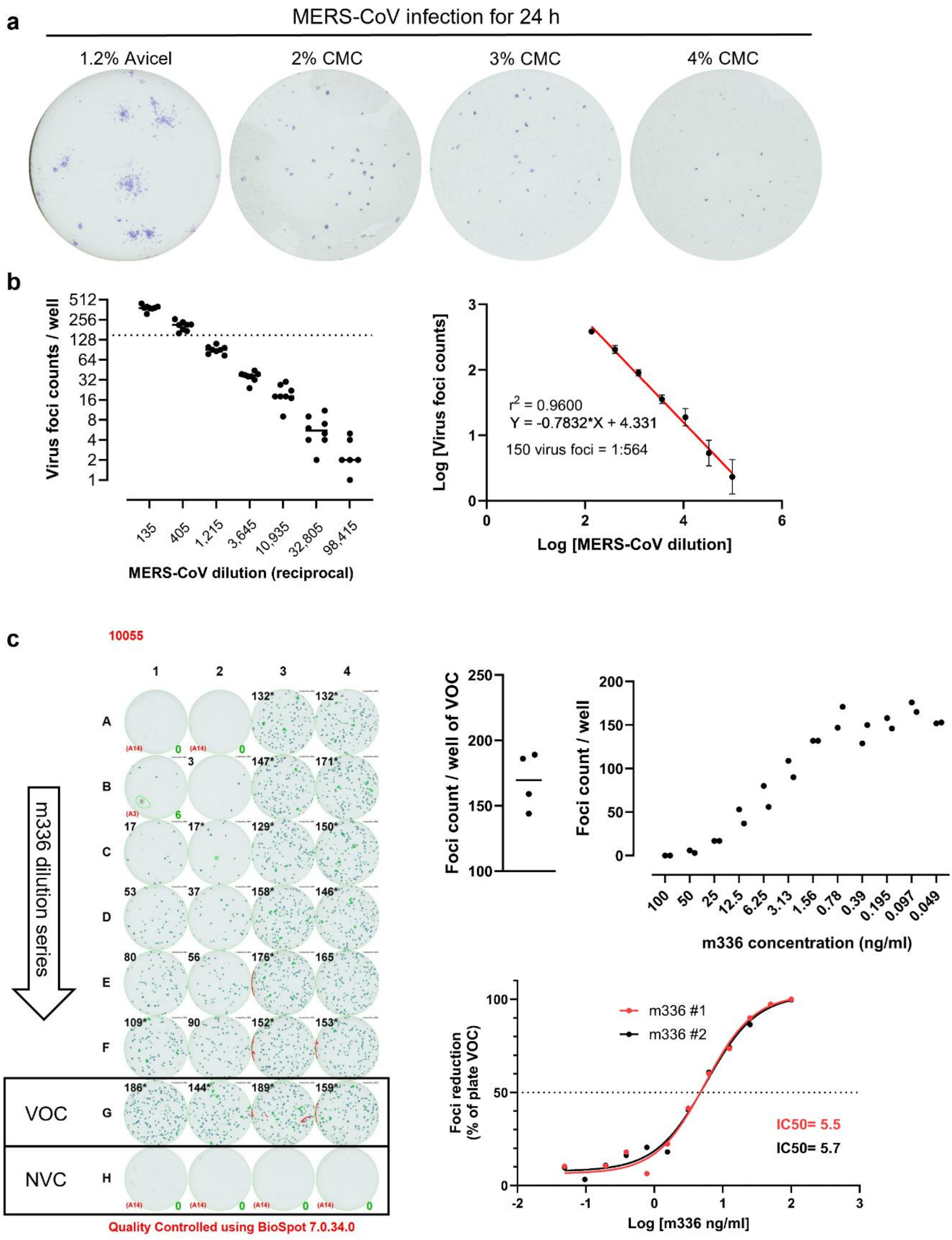

Supplementary Fig. 2 Microneutralization assay establishment

a) Vero C1008 cells were infected with MERS-CoV for 1 h. Then the inoculum was removed and different solutions containing either 1.2% Avicel or different concentrations of carboxymethyl cellulose (CMC) were added. After 24 h incubation, cells were fixed with 4% paraformaldehyde and stained with anti-N-MERS-CoV and peroxidase-coupled antibodies. The resulting virus foci were detected and

analyzed. **b)** Vero C1008 cells were infected with a serial dilution of MERS-CoV as described in A) (diluted 1:3 starting with a 1:5 dilution; n = 8). 2% CMC was used as an overlay. The number of virus foci per well is shown for the corresponding virus dilutions. Linear regression analysis was performed to interpolate the dilution that can be used to generate approximately 150 virus foci per well. **c)** First analysis of an m336 dilution series (1:2 in duplicate) starting at a concentration of 100 ng/ml (from A1, A2 to F1, F2 and then further from A3, A4 to F3, F4.). The number of foci per well were determined by using ImmunoSpot S6 Ultra-V Analyzer (CTL, S6ULTRA-02-6147/ Series 5). The foci reduction compared to the VOC (mean of 4) was calculated for each antibody concentration. These values were used to interpolate 50% inhibitory concentration (IC_{50}) of m336 by four-parameter logistic (4PL) regression analysis. The analysis was performed in duplicate on the same 96-well plate (m336 #1 and m336 #2). The results are shown in black and red.

		MNA	VNT_{100}
**Cells**	**Type**	Vero C1008	HuH7
	**Addition**	Cell monolayer, seeded the day before	Added in suspension to serum dilutions
	**Number / 96 well**	22.000	~10.000
**Sera**	**Initial dilution (LLOD)**	1:20 (20)	1:8 (8)
	**Replicates**	2	3 + 1 to control for toxic effects of each serum
	**Amount required**	24 µl	100 µl
	**Dilution range (dilution series)**	1:20 – 1:4680 (1:3 dilution series)	1:8 – 1:1024 (1:2 dilution series)
	**Number per plate**	6	4
	**Number analyzed in the same working time**	24	80
**MERS-CoV**	**Amount**	Dilution to generate 150 virus foci	100 PFU
	**Determination of virus dilution**	In the assay format (Vero C1008 cells)	Titration of the stock virus (HuH7 cells)
	**Virus quantity used corresponds to**	2000 PFU	100 PFU
	**Incubation time**	24 hours	Four days
	**Titration (stock virus)**	Plaquetest<br>Vero C1008<br>(24 well format)<br>Incubation time 48 h<br>Overlay 1.2% Avicel	Plaquetest<br>HuH7 cells<br>(24 well format)<br>Incubation time 48 h<br>Overlay 1.2% Avicel
**Evaluation**	**Visualization of virus replication**	Specific immunostaining of virus foci	Cytopathic effect (CPE)
	**Readout method**	Semi-automated counting of virus foci	Microscopic evaluation of CPE
	**Result**	Concentration / reciprocal neutralizing titer inhibiting MERS-CoV to 50% (IC_{50} / NT_{50})	Reciprocal neutralizing titer inhibiting MERS-CoV to 100% (VNT_{100})
	**Documentation**	Plate scan (spot reader)	By operator

Supplementary Table 1 Comparison of main characteristics of the two neutralization tests

The most important features relevant to the assays are listed, such as cells, serum dilutions, virus and titration analysis, and the evaluation of both methods. Microneutralization assay (MNA), virus neutralization test (VNT), lower limit of detection (LLOD), Tissue culture infectious dose 50% ($TCID_{50}$) plaque forming units (PFU), cytopathic effect (CPE). For further information about the tests, please contact us directly.
